# Chitosan-Graphene Oxide 3D scaffolds as Promising Tools for Bone Regeneration in Critical-Size Mouse Calvarial Defects

**DOI:** 10.1038/s41598-017-16599-5

**Published:** 2017-11-30

**Authors:** Anca Hermenean, Ada Codreanu, Hildegard Herman, Cornel Balta, Marcel Rosu, Ciprian Valentin Mihali, Alexandra Ivan, Sorina Dinescu, Mariana Ionita, Marieta Costache

**Affiliations:** 1 Department of Histology, Faculty of Medicine, Vasile Goldis Western University of Arad, 86 Rebreanu, 310414 Arad, Romania; 2 Department of Experimental and Applied Biology, Institute of Life Sciences, Vasile Goldis Western University of Arad, 86 Rebreanu, 310414 Arad, Romania; 3 Department of Functional Sciences, Victor Babes University of Medicine and Pharmacy, 300041 Timisoara, Romania; 4 Department of Biochemistry and Molecular Biology, University of Bucharest, 91–95 Splaiul Independentei, 050095 Bucharest, Romania; 5 Advanced Polymer Materials Group, University Politehnica of Bucharest, CaleaVictoriei 147, Bucharest, 010737 Romania

## Abstract

Limited self-regenerating capacity of human skeleton makes the reconstruction of critical size bone defect a significant challenge for clinical practice. Aimed for regenerating bone tissues, this study was designed to investigate osteogenic differentiation, along with bone repair capacity of 3D chitosan (CHT) scaffolds enriched with graphene oxide (GO) in critical-sized mouse calvarial defect. Histopathological/histomorphometry and scanning electron microscopy(SEM) analysis of the implants revealed larger amount of new bone in the CHT/GO-filled defects compared with CHT alone (p < 0.001). When combined with GO, CHT scaffolds synergistically promoted the increase of alkaline phosphatase activity both *in vitro* and *in vivo* experiments. This enhanced osteogenesis was corroborated with increased expression of bone morphogenetic protein (BMP) and Runx-2 up to week 4 post-implantation, which showed that GO facilitates the differentiation of osteoprogenitor cells. Meanwhile, osteogenesis was promoted by GO at the late stage as well, as indicated by the up-regulation of osteopontin and osteocalcin at week 8 and overexpressed at week 18, for both markers. Our data suggest that CHT/GO biomaterial could represent a promising tool for the reconstruction of large bone defects, without using exogenous living cells or growth factors.

## Introduction

The most common approach for bone tissue engineering has attempted to mimic the natural process of bone repair by delivering a source of progenitor cells capable for osteoblastic differentiation. These requirements can be provided by three dimensional biocompatible, bioresorbable and osteoconductive scaffolding matrices, which support *in vivo* cellular attachment, migration, and proliferation of osteogenic cells^1^.

Three-dimensional porous scaffolds may enhance bone regeneration by representing an appropriate environment which facilitates invasion of cells from surrounding tissues, proliferation, differentiation, development of bone extracellular matrix (ECM) and vascular beds^2^. Potentially porous suitable scaffolds for use in bone regeneration includes ceramics, such as hydroxyapatite, beta-tricalcium phosphate^3^, titanium dioxide^4^ and bioactive glass; synthetic or bio-polymers, such as poly-lactic acid^5^, poly-glycolic acid^6^, polycaprolactone^7^, chitosan or collagen, and composites of the upper mentioned materials *i.e*.ceramic/polymer (hydroxyapatite/chitosan)^8,9^.

Ceramic materials were extensively used as bone tissue engineering substrates, owing to their high strength, tissue compatibility and good osteoconductivity, while biodegradation, difficulty of shaping and fragile mechanical properties are important limits to be considered ideal material for clinical applications^10^. Contrariwise, polymeric-based biomaterials were noticed for their biodegradation properties combined with excellent tissue-compatibility^11,12^, however poor mechanical features or irregular release of osteoinductive factors for many polymer materials were noticed^6^.

One of the most promising polymeric material seems to be chitosan (CHT) a biopolymer, obtained by deacetylation of chitin. It has been reported to be safe, hemostatic, biocompatible, osteoconductive, promoter of mineralized bone matrix^13^, and provider of a minimum inflammatory response after implantation^14^.

In the last years, graphene oxide (GO) has attracted much interest for uses in bone tissue engineering^15^, because of its unique physico-chemical and mechanical properties, as well as good biocompatibility^16,17^. The interactions with proteins through hydrophobic and electrostatic interactions^16,18,19^ could potentially enhance the osteogenic differentiation of progenitor cells and promote bone formation. A distinct feature of GO is that can form a uniform and stable suspension in water in contrast with graphene, which tends to form aggregates. Afterwards aqueous GO suspension could infiltrate into the inner parts of a porous scaffold and modify the surfaces of the pore walls^20^, which can be a really advantage for scaffold candidates of bone tissue engineering.

In our previous works, and one of the first studies worldwide, we demonstrated the importance of GO in either bidimensional (2D) or tridimensional (3D) biomaterials composition for *in vitro* cell viability and proliferation. Thus, chitosan/GO composite films with 0.5, 1, 2.5 and 6.0 wt.% GO proved to be biocompatible for MC3T3-E1 murine preosteoblasts, cells adapted faster and proliferated much more in contact with the composite with a higher content of GO^21^. Similar results were obtained for other composite materials based on GO but with different polymer matrix such as chitosan–polyvinyl alcohol films (CS-PVA/GO)^22^, or films based on polysulfone (PS) and GO nanosheets^23^. Furthermore the addition of GO to the tridimesional composite scaffold of the CHT/GO increased proliferation profile of MC-2T3 murine preosteoblasts after 7 days of direct contact with materials and the extracts released by these composites in the surrounding environment exhibit no significant cytotoxic effects after 24 h of seeds^24^. In other study, gelatin-poly(vinyl alcohol) biocomposites reinforced with GO provided equilibrated physico-chemical properties and low cytotoxic profile, that allowed murine preosteoblasts viability^25^.

The potential advantages of using GO-based CHT scaffolds directly as factors inducing cellular differentiation, as well as bone tissue regeneration are not been explored.

The objectives of the present study were to create highly porous three dimensional chitosan/graphene oxide (CHT/GO) composite scaffolds and to evaluate the *in vitro* and *in vivo* osteogenic effects with GO addition to biomaterial composition. Bone formation, bone maturation, bone distribution and mineralization were studied by histology, histomorphometry and scanning electron microscopy. The osteogenic differentiation of progenitor cells, osteoblast differentiation and proliferation were analyzed quantifying specific protein expressions and osteogenic gene by using immunofluorescence and RT-PCR assays.

## Results

### *In vitro* results

#### Alkaline phosphatase (ALP) activity during *in vitro* differentiation studies

*In vitro* osteogenic differentiation studies were performed in order to determine CHT/GO biomaterials potential to support cell differentiation processes on short term, in the view of possible implantation for bone tissue engineering purposes.

**Figure 1:**
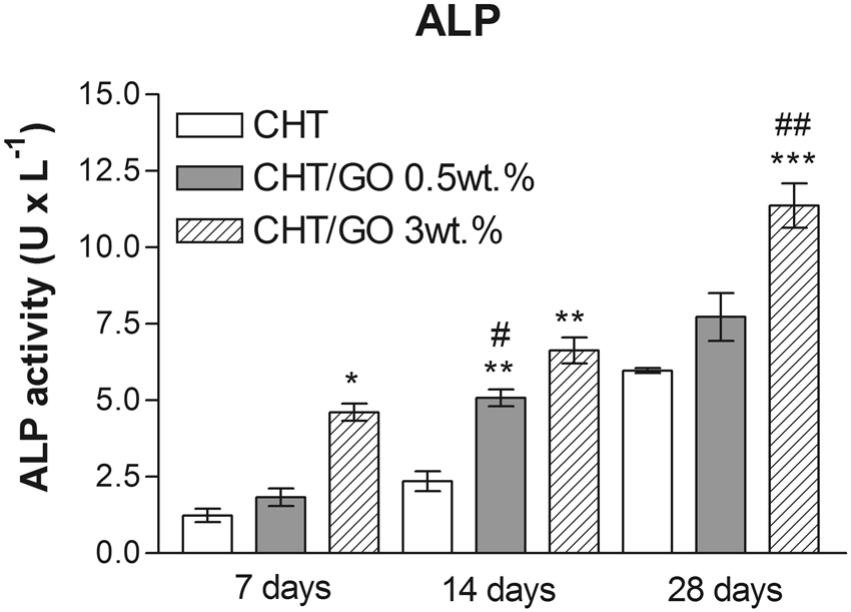
Spectrophotometrically determined ALP activity in cells undergoing osteogenic differentiation in CHT/GO biomaterials during 28 days of *in vitro* culture.

ALP is a common molecular marker for osteogenic differentiation, thus its activity was relevant to assess the evolution of the induced osteogenic process in our experimental conditions. After 7 days of differentiation, ALP levels clearly proved the activation of osteogenesis in all samples (Fig. 1). In particular, ALP activity was significantly increased in cells cultured in CHT/GO 3 wt.% (p < 0.05), as compared to the control CHT. This significant increase in ALP was also found after 14 (p < 0.01) and 28 days (p < 0.001) of differentiation, suggesting a positive contribution of 3 wt.% GO to the efficiency of osteogenic differentiation process. Interestingly, the effect of CHT/GO 0.5 wt.% material on cell osteogenic differentiation was not as constant and remarkable as of CHT/GO 3 wt.%- after 14 days, ALP activity registered an important increase (p < 0.01) as compared to the control (CHT), whereas after 28 days of differentiation this difference in ALP activity related to the control was not statistically visible anymore, thus suggesting that the addition of a low percentage of GO to material composition could have an impact on cell osteogenic differentiation, but unstable on a long term.

### *In vivo* results

The osteogenic potential of CHT-GO scaffolds were tested *in vivo* using a critical bone defect model in the mouse calvaria. The follow-up was performed 72 hours, 4, 8 and 18 weeks post-implantation.

#### ALP activity

**Figure 2:**
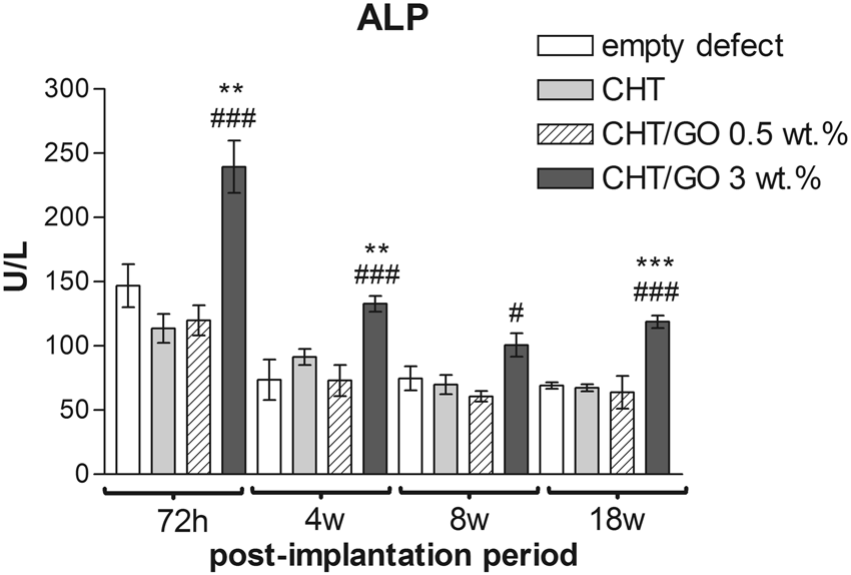
ALP activity in mice calvaria defects implanted with CHT/GO after 7 h, 4 weeks, 8 weeks and 18 weeks post-implantation.

As shown in Fig. 2, the level of ALP has increased after 3 days post-implantation. ALP activity analysis revealed higher level of ALP on CHT/GO 3.0 wt.% than on CHT (p < 0.001) and control (p < 0.01) at 72 hours post-implantation. Even at 18 weeks, the level of ALP on CHT/GO 3.0 wt.% is significant higher than in CHT and control (p < 0.001).

#### Histological findings

**Figure 3:**
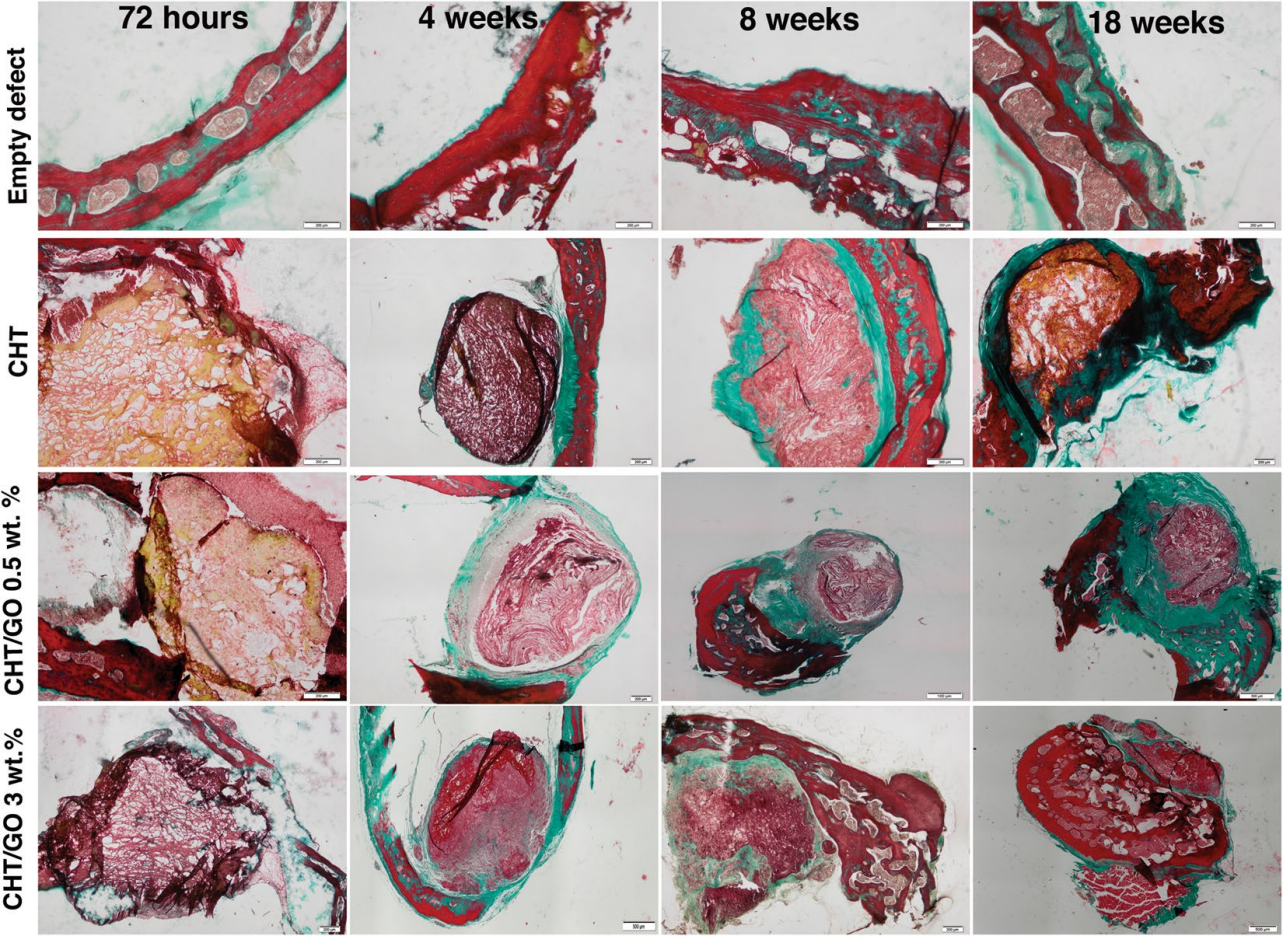
Histology of the repaired calvaria after 72 hours, 4, 8 and 18 weeks of CHT-GO scaffold’s implantation. *Images from MassonGoldnertrichrome staining*. *Empty defect group*: At weeks 4, 8 and 18, the area between the surgical bone margins was observed to be filled with a thin, loose connective tissue. *Group CHT:* At week 4 defect sites were filled with dense connective tissue, containing a scarce amount of inflammatory cells, fibroblasts, and few blood vessels. Fibrous connective tissue was observed surrounding the scaffold. Most of the CHT did not appear to have been resorbed. *Group CHT/GO 0.5 wt.%*: This group showed more fibrous tissue infiltration at weeks 4 and 8 compared with CHT group. New bone formation was observed outlying from the surgical margins, while osteoid tissue and calcified bone spicules appearsneighboring connective tissue at week 18. The CHT/GO 0.5 wt.% material was most resorbed than CHT alone after 18 weeks post-implantation, but large fibrous capsule was observed. Group *CHT/GO 3.0 wt.%*: A favorable evolution of the repair process was observed in presence of CHT/GO 3.0 wt.%. Scale: 200 µm.

Qualitative histological observation of thin sections of the calvarial defects at 18 weeks post surgery showed that the bone defects filled with CHT-GO scaffolds, had superior healing compared to both untreated and chitosan scaffold-treated defects. New bone formation in the CHT scaffolds containing GO 3.0 wt.% occurred into the periphery and along the center of the implants. The quantity of newly formed bone in the CHT/GO 3.0 wt.% grafts was substantially higher at 18 weeks than that in the CHT grafts, showing more advanced stages of remodeling and consolidation (Fig. 3).

**Figure 4:**
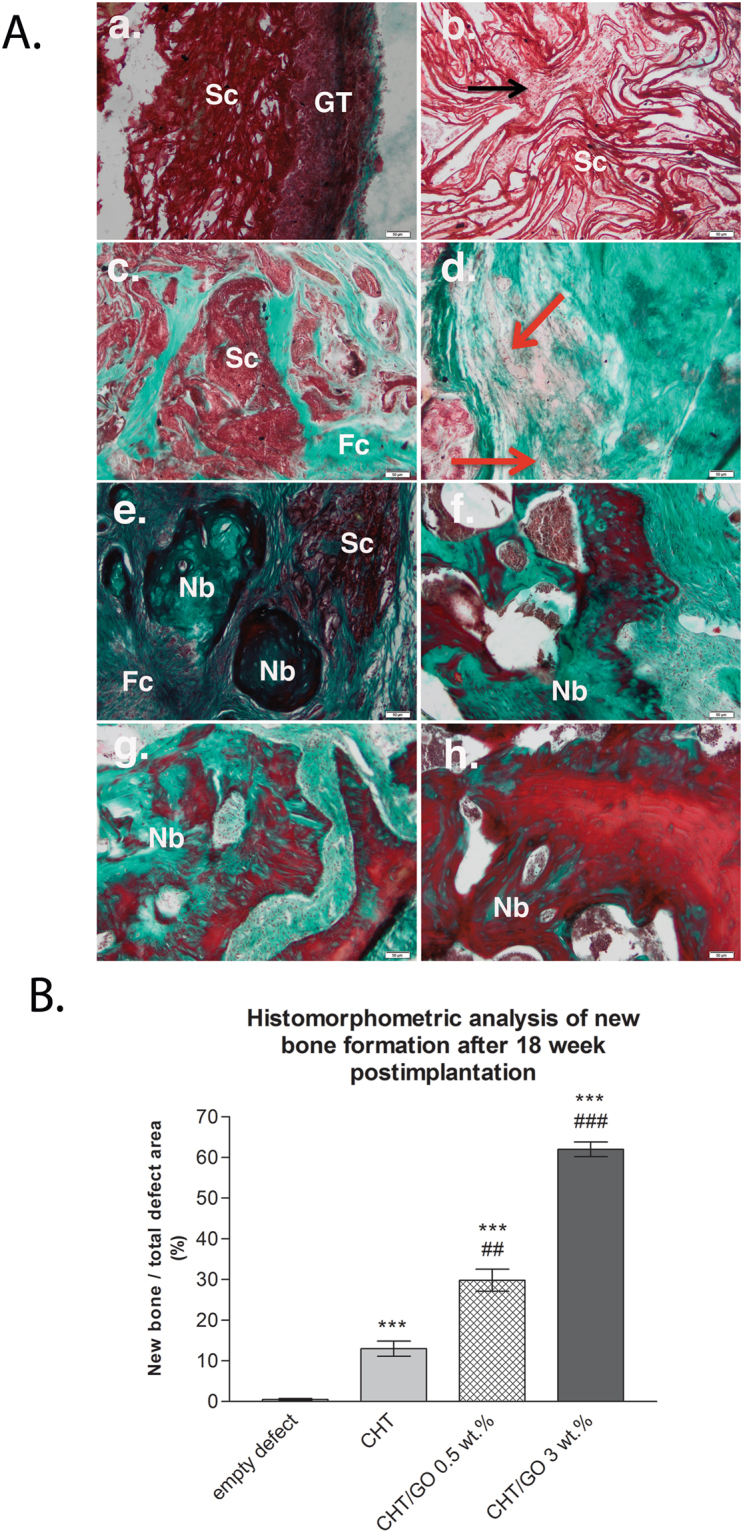
(**A**) Histology detail of the repaired calvaria after 3 days, 4, 8 and 18 weeks of CHT/GO 3.0 wt.% implantation (a) and (b) *3 days post-implantation*; (c) and (d) *4 weeks post-implantation*; (e) and (f) *8 weeks post-implantation*; (g) and (h) *18 weeks post-implantation*; symbols: scaffold (Sc); granulation tissue (GT); fibroconnective tissue (Fc); capillary (red arrow); new bone island (Nb); osteoprogenitor cells infiltration (black arrow); Masson Goldnertrichrome stain. Scale 50 µm. (**B**) Histomophometric analysis of Masson Goldner trichrome-stained sections showing total bone regeneration in mice calvarial defects implanted with CHT/GO after 18 weeks post-implantation.

Higher-magnification images of the Masson Goldner trichrome-stained sections of the defects implanted with CHT/GO 3.0 wt.% are shown in Fig. 4A.

At 72 hours post-surgery CHT/GO 3.0 wt.% led to an increased number of cells related with granulation tissue (GT) infiltration from the periphery to the defect center (Fig. 4A). Fibrovascular tissues and biomaterial resorption were increased compared with other two scaffolds, indicating that the CHT/GO 3.0 wt.%, after it had been implanted for 4 weeks, accelerated the restoration of defective bone. Eight week after surgery, histological evaluation indicated progressive growth of more newly formed bone from the calvaria margins toward the center of the bone defect. Furthermore, CHT/GO 3.0 wt.% appear well-preserved and in intimate contact with surrounding bone, without visible fibrous capsule. At week 18, new bone island were observed near the surgical bone margins, around and within graft material. The newly formed bone was mostly woven bone, enriched in collagen (green stained fibers). In other areas, we found normal architecture for new bone (Fig. 4A).

Histomorphometry revealed a significantly higher percentage of new bone area after 18 weeks in the experimental groups than in the empty defect group (Fig. 4B). However, there was significant difference between CHT/GO 3.0 wt.% group and empty defect, or control CHT group (p < 0.001).

#### Scanning Electron Microscopy (SEM) analysis of *in vivo* samples

**Figure 5:**
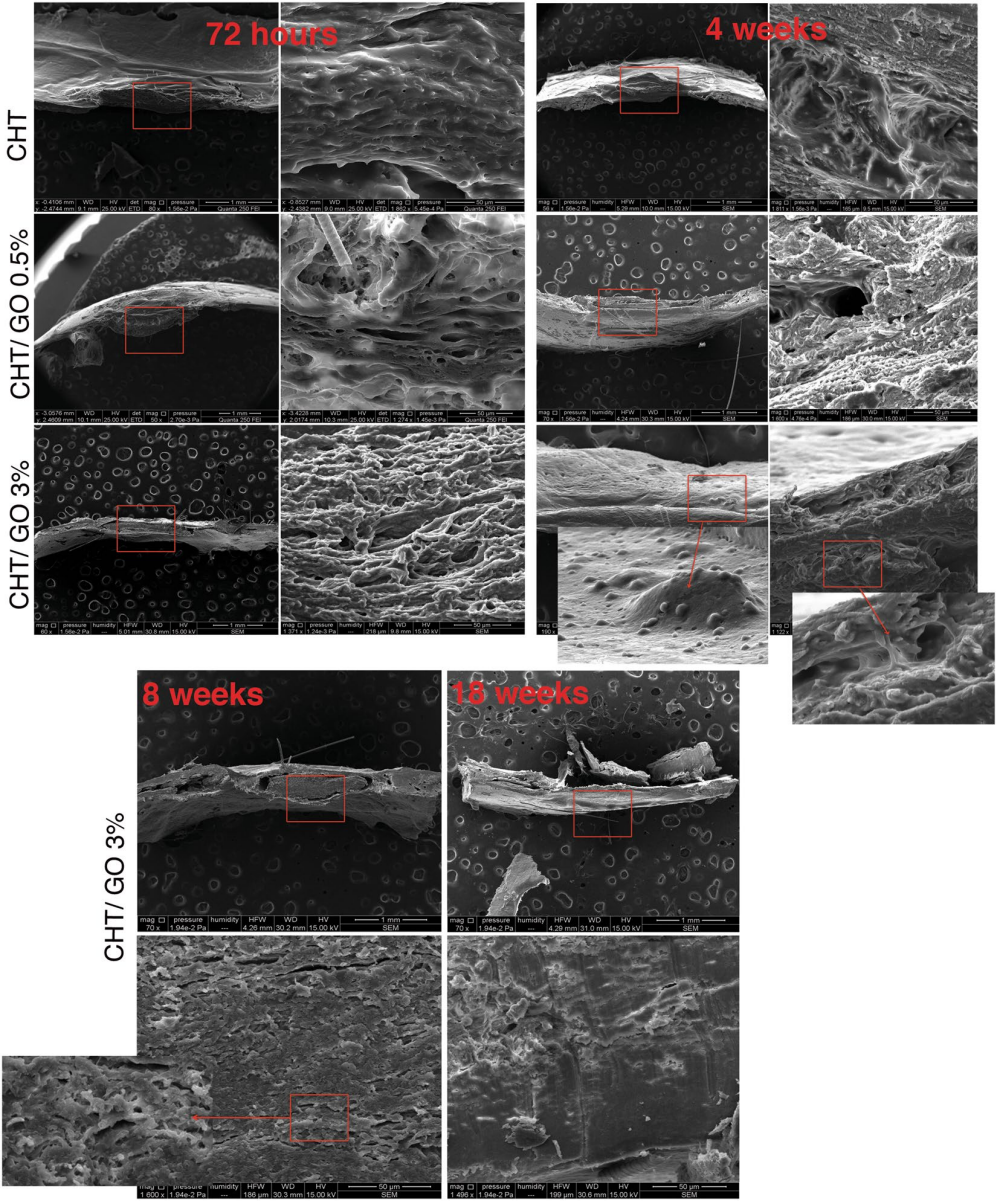
SEM micrographs of the *in vivo* bone samples taken after 72 hours, 4, 8 and 18 weeks after CHT, CHT/GO 0.5 wt.% and CHT/GO 3.0 wt.% scaffold implantation.

Figure 5 reproduces SEM micrographs taken from the implant area. At 72 hours after implantation, scaffolds begin to populate with cells, which is evident after 4 weeks when osteogenic cells were found on the surface and within the bulk of the scaffolds, especially for GO 3.0 wt%. Furthermore deposits of calcium phosphate crystals were observed indicating that cells start to produce ECM after 4 weeks from implantation. The micrograph confirm mineralization of CHT/GO 3.0 wt.% implant over week 8 and at week 18 there is no difference between new bone and host bone.

#### *In vivo* osteogenic differentiation

In order to examine the osteogenic differentiation of bone cells on CHT/GO 3.0 wt.% scaffold, we analyzed the expressions of early and late osteogenic markers by RT-PCR and immunofluorescence, after 72 hours, 4, 8 and 18 weeks post-implantation.

**Figure 6:**
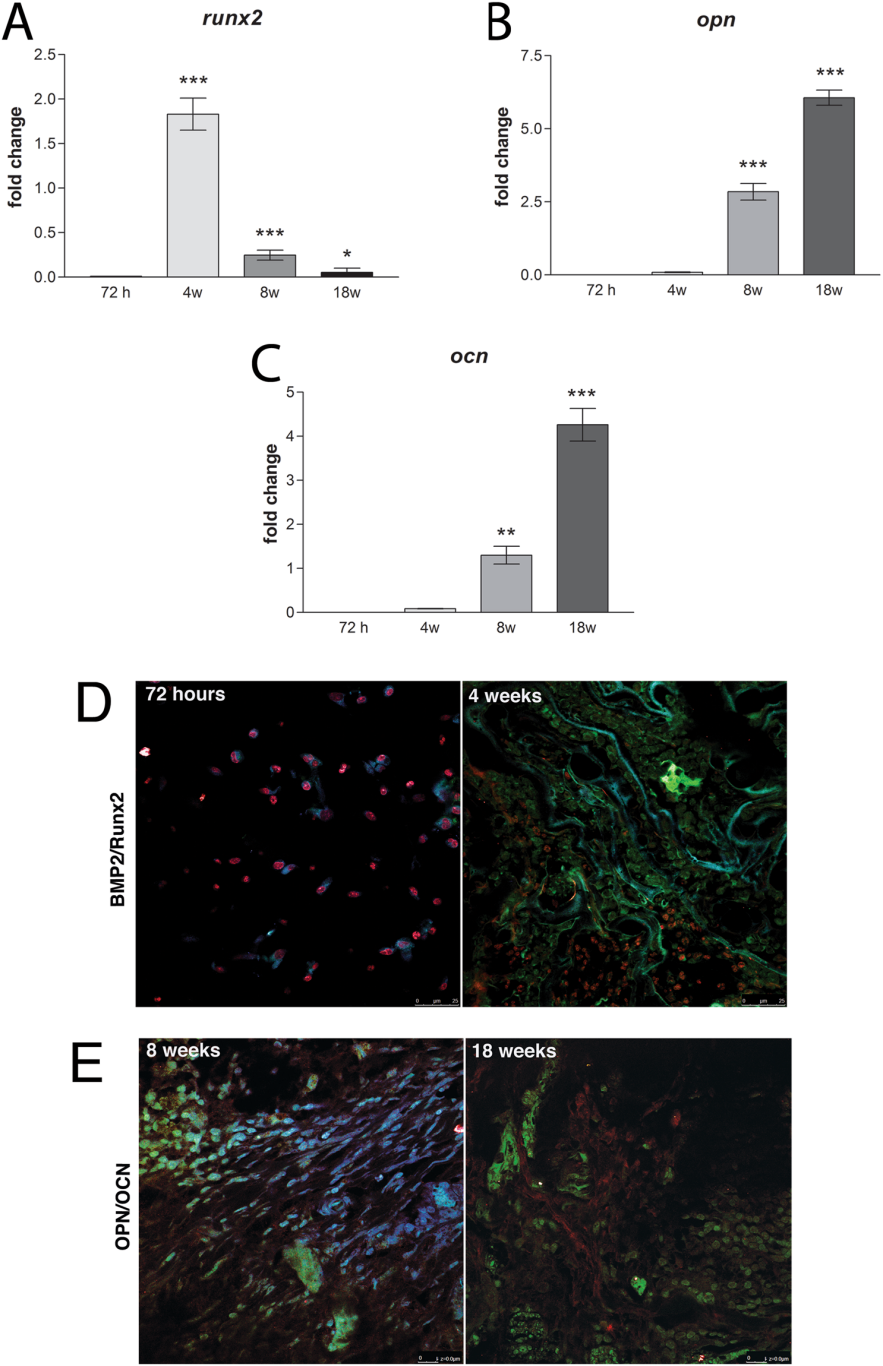
CHT/GO 3.0 wt.% implantation stimulates early and late osteogenesis markers. (**A**) mRNA expression of Runx-2 at 72 h, 4 weeks, 8 weeks and 18 weeks pos-timplantation; (**B**) mRNA expression of OPN at 72 h, 4 weeks, 8 weeks and 18 weeks post-implantation; (**C**) mRNA expression of OCN at 72 h, 4 weeks, 8 weeks and 18 weeks post-implantation; (**D**) Immunohistochemical expression of BMP-2 and Runx-2 at 72 h and 4 weeks post-implantation; (**E**) Immunohistochemical expression of OPN and OCN at 8 and 18 weeks post-implantation.

Runx-2 is an early osteogenic marker. Gene expression studies performed in mice revealed Runx-2 activation (Fig. 6A) and a maximum of expression at 4 weeks post-implantation (p < 0.001). Once the process was induced in the tissue surrounding CHT/GO 3.0 wt.% implant, Runx-2 levels significantly decreased up to 8 weeks of study (p < 0.001) and reached the basal incipient level by the end of experiment (18 weeks). This profile is typical for a gene with early activation in bone differentiation process and confirms the ability of CHT/GO 3.0 wt.% material to support and efficiently maintain bone regeneration at the implantation site.

Furthermore, late osteogenic markers typical for bone matrix OPN and OCN registered an increasing expression profile during 18 weeks of *in vivo* bone regeneration using CHT/GO 3.0 wt.% (Fig. 6B,C). Both OPN and OCN registered statistically significant increase in gene expression 8 weeks post-implantation (p < 0.001 and p < 0.01, respectively). Conversely, 18 weeks post-material implantation, OPN revealed a 7.5-fold difference in gene expression related to the moment of implantation, whereas OCN registered a 5-fold difference, both levels of expression being statistically significant increased (p < 0.001) as compared to 8 week levels.

Immunofluorescence targeting BMP, Runx-2, OCN and OPN was performed. BMP and Runx-2 resulted in a strong staining in all tissues (bone and connective tissue that had infiltrated the scaffolds), however a more intense staining was found at the location of bone formation in week 4 (Fig. 6D). OCN was expressed in the bone matrix and was more intense 8 weeks post-implantation (Fig. 6E). Osteopontin was mainly expressed in the osteoblast lacunae and at the ossification line as well and has been rising gradually to 18 weeks (Fig. 6E).

## Discussion

The keypoint to successful bone generation is to provide the repair site with sufficient osteoprogenitor cells in a suitable delivery biomaterial, in order to insure osteoblastic differentiation and optimal secretory activity. Porous biomimetic materials may provide a suitable microenvironment that promotes osteoblast proliferation and osteogenesis^1^.

In order to address this issue, we aimed to determine *in vitro* and *in vivo* osteogenic differentiation and bone regeneration capacities of highly porous three dimensional chitosan composite scaffolds enriched with different concentration of graphene oxide in critical-sized mouse calvarial defect.

There is an open question about the size of bone defects in rodent calvarial bone. It is well known that for regeneration of large bone defects, is important to develop bioactive scaffolds, with distinct properties of promoting osteogenesis and inducing the formation of new bone *in vivo*. It is important to ensure that bone defect in the empty defect group does not heal spontaneously during the study, so as in our empty defect group, the defect was not healed by the end of the eighteen weeks. Our choice was based on the previous knowledge that 4–5 mm size of the defect in rodent calvaria is sufficient for evaluating new bone formation within timeframe between 4 and 24 weeks^26–30^.

Based on a biomimetic approach, we have specifically designed a porous three dimensional chitosan-graphene oxide composite scaffold, which combines biocompatibility provided by chitosan with exceptional physical properties of graphene oxide and exhibits improved mechanical properties, appropriate pores architectures, structural features, cell proliferation and viability to support new bone formation^24,31,32^.

The positive effects of chitosan on bone healing have been demonstrated in several *in vitro* and *in vivo* studies^33^, while graphene oxide has been used to enhance bone healing and to accelerate the regeneration and osteointegration of biomaterials, such as hydroxyapatite^34^, ß-tricalcium phosphate^20^, titanium implants^35^. However, to the best of our knowledge, the combined effect of chitosan and graphene oxide on bone defects has not been explored *in vivo* before.

In our study, to evaluate bone formation, the 5 mm mouse critical-size defect was used. The results of the present study showed that the incorporation of 3.0 wt.% GO in the chitosan scaffolds significantly enhanced bone regeneration in mice calvarial defects at 18 weeks when compared to the CHT scaffolds (p < 0.001) (Fig. 4), even in the absence of exogeneous differentiating agents.

Histological observations at 18 week post-implantation in the empty defect group showed limited bone healing close to bone defect border, with a thin connective tissue bridging. This verified the incapacity of the endogenous mechanisms to fully regenerate defects of this size, without exogenous interference. In chitosan-treated defects, bone healing was only slightly higher to that of the control group, confirming the restricted osteogenic properties of the chitosan biomaterial alone. Moreover, as indicated by the histology and SEM results, chitosan scaffold showed poor osteoblast proliferation, which is in agreement with previous study^36^. Possible reasons for this lower healing percentage (13.00 ± 4.18%) of chitosan in our study is that the scaffold was not degraded properly, biodegradation depending on different factors, *e.g*., degree of crystallinity, water content, the shape/condition of the surface on the material^37^ and/or intrinsic hydrolases mechanisms^38^. In our previous studies we have demonstrated that addition of GO within CHT matrix produces changes in both structure and morphology (pores architectures). Regarding the morphology, we found that the addition of GO to CHT promotes the formation of scaffolds with highly interconnected porosity and well defined pores, while the pure CHT displays a rough morphology with undefined pores^32^. It is the authors opinion that the rough morphology with undefined pores of pure CHT scaffold is responsible on the one hand for to lower migration of the cells within the scaffold and on the other hand for lower penetration of body fluids and low rate of material degradation. Histological analysis of the chitosan improved with graphene oxide groups revealed that newly-formed bone was increased from the periphery to the center of the bone defect with graphene oxide concentration, rather than only at the border, as was observed in chitosan group. The difference in bone infiltration in the two types of scaffolds (CHT and CHT/GO) may result from differences in the scaffold architecture. Biomaterial porosity, pore size and pore interconnectivity, is well known to play a key effect on bone infiltration^39^. Our previous study, revealed that the addition of graphene oxide into the polymer matrix led to a slight decrease of interconnected pores size. On the other hand GO - free scaffold reveals undefined pore shapes, with smooth and thick pore walls, while a more homogenous architecture is obtained with the incorporation of GO within CHT matrix and is leading to thinner yet more crumpled pore walls at the same time which allow easier cell infiltration, attachment, proliferation, differentiation, ECM development and integration with host bone^24^.

In the same time, biodegradability noticed by histological observation and *in vivo* lower inflammatory response of CHT/GO3.0 wt.%^40^, contributes to increased bone healing abilities, comparing with CHT/GO0.5 wt.% or CHT, and makes it one of the promising scaffolds for osteogenic applications.

A key component of this study was to evaluate the *in vivo* osteogenic capacity of CHT/GO scaffolds, compared with CHT alone and control one. It was also observed that the cell infiltration and vascular invasion into scaffolds is controlled by two keys factors: time frame and GO presence, *i.e*. the infiltration increasing over the duration of the experiment and with GO concentration. These findings indicate that cell migration into CHT/GO scaffolds and overall cell distribution can be enhanced by particular porosity, pore-size and physico-chemical properties provided by graphene oxide addition, which can influence cell attachment and infiltration, support cell proliferation and direct osteoblastic differentiation. Besides, we have shown *in vitro* biocompatibility of CHT/GO 0.5 wt.% and CHT/GO 3 wt.%, in terms of cell metabolic activity and proliferation, than CHT control, but with different rates p < 0.05 and p < 0.001, respectively^24^.

The advantageous porosity and interconnectivity of our scaffold^24^ are supported by the findings in that it permitted excellent vascularization in all biomaterial area. This is of significance as bone formation is closely connected to the level of vascular network^41^ and is a necessary phase for bone in growth process. In this study we observed the formation of microvessels initially at the periphery of the CHT/GO scaffolds, where the bone was formed first, indicating that the newly formed bone was not deprived of oxygen and nutrients^7^. The microvascularisation in the reparative area provided pluripotent perivascular cells that were capable of differentiating into osteoblasts.

Osteogenesis is a complex process with a number of factors, which are involved in the stimulation of osteoblastic differentiation. In the regulation of osteogenesis, many signaling pathways are involved in, such as the MAPK, BMP, Wnt, and NF-kB signaling^42^. Although much is known about how early and late osteogenesis markers interfaces with the regulatory signaling pathway *in vitro*, less is known about how they interact during bone development and repair *in vivo*, while none results were previous recorded for CHT/GO scaffolds as osteogenic promoters in living organisms.

In the present study, we observed several GO-specific effects on osteogenesis and bone repair process. During the early proliferation period (3 days), BMP positive staining was observed directly within the scaffolds enriched with graphene oxide, with a maximum peak at week 4. This is of importance, because BMP-2 markedly up-regulates the expression of Runx-2 transcriptional factor through the activation of Smad signaling, thereby stimulating osteogenic differentiation of mesenchymal stem cells^43,44^. Furthermore, Smad1 and Smad5 physically interact with Runx-2 and enhance its transcriptional and osteoblastogenic activity^45^, which is consistent with our findings. The maximum up-regulation of Runx-2 activity obtained at week 4 with the presence of GO (Fig. 6), suggested that osteogenesis of chitosan scaffolds was improved by GO at the early stage.

At the same time, the ALP activity, marker of early osteogenic differentiation, was also enhanced by GO, both *in vitro* and *in vivo* experiments (Figs 1 and 2). These data suggest that GO facilitates the differentiation of osteoprogenitor cells to an osteogenic lineage.

Meanwhile, osteogenesis was promoted by graphene oxide at the late stage as well, as indicated by the upregulation of OPN and OCN at week 8 and overexpressed at week 18, for both markers. CHT/GO scaffolds showed a very significantly higher transcript level compared to CHT alone or even to the normal osteogenic control. The up-regulation of osteoblast-specific OPN and OCN gene expression is consistent with the change in Runx-2 mRNA and nuclear protein expression levels in response to GO addition, which implies that GO modification of osteoblast gene expression is a reflection of Runx-2 expression modulation, since OPN and OCN are regulated by Runx-2 (Fig. 6).

In the present study, group CHT/GO 3 wt.% presented a significant increase in the number of OPN and OCN-positive cells when compared to CHT/GO 0.5 wt.%, or CHT alone. It reveals that the increase in graphene content enhance the induction of OCN and achieves higher surface area to promote the cell attachment, proliferation, maturation, and finally matrix mineralization^46^. The new bone synthesis is directly dependent on the secretion of osteocalcin in the cells, while mineralized extracellular matrix contains smaller but significant amounts of osteocalcin^47^.

## Conclusions

The pattern of early and late osteogenesis markers expression and the values of related gene expression observed in mouse model group CHT/GO 3 wt.%, as well as histopathological and SEM observations, suggests that this group had the most developed healing process, when compared to CHT/GO 0.5 wt.%, or CHT alone.

Our data suggest that CHT/GO biomaterial could represent a promising tool for the reconstruction of large bone defects, without using exogenous living cells or growth factors.

## Materials and Methods

### Materials

Graphene oxide used for scaffolds fabrication was obtained according to Hummers procedure and supplied by National Institute for Research and Development in Microtechnologies (Romania) (William, Hummers, & Richard, 1958). Medium molecular weight Chitosan from crab shells and Acetic acid (≥99.7%) were purchased from Sigma-Aldrich. All materials were used without further purification and the water used in this work was double distilled water.

#### For *in vitro* study

Osteogenic differentiation kit was provided by Thermo (StemProOsteogenic Differentiation kit, Gibco, Thermo Fisher, Foster City, CA), while for ALP assay was used colorimetric Alkaline Phosphatase Assay Kit (Abcam).

#### For *in vivo* study

ALP assay kit was obtained from ChemaDiagnostica, Monsano, Italy. Histopathological reagents and staining kits were purchased from BioOptica (Italy) and bone morphogenetic protein (BMP), Runx-2, Osteocalcin (OCN), Osteopontin (OPN), all from Santa Cruz Biotechnology (USA). RNA isolation kit (InnuPrep RNA Mini Kit) was obtained from AnalytikJena, (Berlin, Germany), Trizol from Life technologies, Thermo Fisher (Foster City, CA) and iScriptcDNA Synthesis kit from BioRad, (Hercules, CA, USA).

### Fabrication of CHT/GO 3D porous scaffolds

CHT/GO scaffold with 0, 0.5, and 3 wt.% GO were prepared by a simple protocol followed by freeze-drying method^24^. Initially the chitosan was mixed in portions under constant stirring at ∼50 °C with appropriate volume of acetic acid solution (10% by weight in water) in order to form homogenous viscous solution of 1wt.% concentration. Thereafter, different contents of graphene oxide flakes (0.5 and 3 wt.%) were added into Chitosan solution and dispersed through ultrasound treatment for 1 h in an ice bath. The well homogenized CHT/GO solutions were casted onto transparent glass Petri dish and frozen over the night at −70 °C. Scaffolds in their final states were obtained by freeze-drying for 2 days at −50 °C (0.040mbar). The 3D dried scaffolds were thermal treated *in vacuum* according to the following protocol: 30 min 50 °C, 30 min 70 °C and overnight at 90 °C.

Ultrasound treatment was performed using a VCX750 ultrasonic processor from Sonics & Materials, Inc. (53 Church Hill Road, Newton, CT 06470-1614 USA) equipped with a titanium alloy (Ti-6Al-4V) probe tip and a 750 W source operating at a frequency of 20 kHz. During the ultrasound treatment the probe tip vibrations were set to 80% amplitude and sonication time was chosen to be 1 h for GO dispersion in CHI 1 wt.% solution.

The freeze-drying techniques was performed at −50 °C and 0.040 mbar, using a Christ LCG Alpha 2–4 LD plus laboratory freeze-dryer equipped with a PMMA chamber (Martin Christ, Gefriertrocknungsanlagen GmbH, Postfach 1713, 37507 Osterode am Harz).

### ALP activation during *in vitro* studies on CHT/GO scaffolds

The novel CHT/GO scaffolds have been tested for their ability to support osteogenic cell differentiation during *in vitro* studies. Briefly, murine preosteoblasts belonging to 3T3-E1 cell line have been seeded in CHT/GO 0.5–3 wt.%, as well as in the reference scaffold CHT. The 3D cell-scaffold systems were exposed to osteogenic conditions (StemProOsteogenic Differentiation kit) for up to 28 days and the evolution of the differentiation process was monitored via alkaline phosphatase (ALP) levels after 7, 14 and 28 days.

ALP activity was evaluated by colorimetric methods from cell culture supernatant samples collected during the *in vitro* differentiation process, following manufacturer instructions (colorimetric Alkaline Phosphatase Assay Kit). All experiments were performed in triplicate.

### Mouse calvarial defect model for evaluating bone regeneration

**Figure 7:**
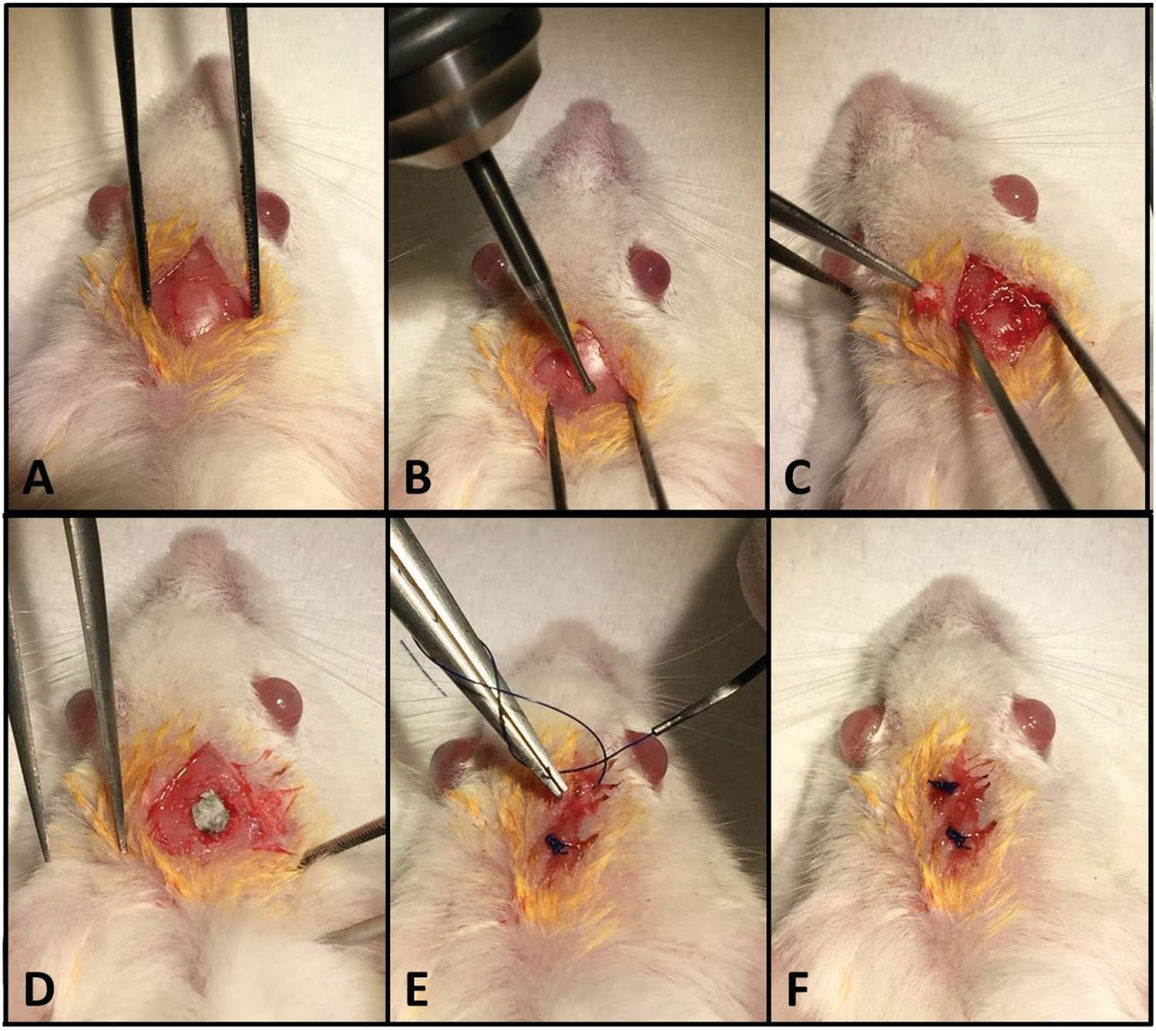
Surgical procedure. (**A**) Preparation of the surgical defect. (**B**,**C**) Execution of the 5-mm critical size defect in the rat calvarium. **(D)** Placement of the chitosan–graphene oxide scaffold (**E**,**F**) Closure of the periosteum and overlaying skin.

CD1 mice (4 weeks old) were used for the experiments. Animal care was in compliance with the Guide for the Care and Use of Laboratory Animals at Vasile Goldis Western University of Arad. All procedures were performed under the supervision and approval of the Ethics Committee for Research of the Vasile Goldis Western University of Arad. One hundred and sixty male mice were enrolled in the calvarial bone defect experiment. The animals were placed under anesthesia during the critical bone defect surgery by intraperitoneal (i.p.) administration of 100 mg/kg b.w.ketamine hydrochloride and 10 mg/kg b.w.xylazine hydrochloride. After anesthesia, calvaria’s were exposed by linear incision in the skin, the periosteum was detached from the bone of the cranium by scraping (Fig. 7). 5-mm full-thickness craniotomy defects were prepared using a 3.5 mm power drill (Super NP5, Korea) under constant phosphate buffer solution (PBS) irrigation, as previously described^48,49^. The periosteum was reflected over the defect site, and the incision was closed with 5.0 nylon sutures and post-operational care was performed. No lethality was detected during the surgery or over the post-surgical period. After surgery, the animals were housed individually under constant conditions.

The mice were randomly divided into three groups: Group A, empty defect; Group B, chitosan scaffolds implanted; Group C, chitosan—0.5wt.% graphene oxide implanted; Group D, chitosan—3.0 wt.% graphene oxide implanted. All the 3D scaffolds were sterilized by ultraviolet radiation (UV) for 20 min.

The animals were euthanatized after 72 hours, four, eight, eighteen weeks (n = 10 for each group at each time point), using an overdose of xylazine-ketamine and the implants were harvested for subsequent evaluation.

### *In vivo* ALP activity

Blood samples were harvested by cardiac puncture and collected into sterile containers without anticoagulant. Biochemical analysis were carried out to determine the serum concentrations of alkaline phosphatase (ALP) using a Mindray BS-120 Chemistry Analyzer (ShenzenMindray Bio-Medical Electronics Co., Ltd., Nanshan, Shenzhen, China).

### Histology and histomorphometry

The calvarial samples consisting of the defect sites with surrounding bone and soft tissue were washed with PBS and fixed in 10% formalin solution for 3 days, washed with water, and sunken in Biodec R (Bio-Optica) for 5 days at room temperature. After decalcification, the samples were dehydrated in ascending concentrations of alcohols, cleared, and embedded in paraffin blocks. Histological sections (5 µm) were prepared using a microtome and subsequently stained with Goldner’s Masson Trichrome for observation of the new bone formation. The stained sections were examined under light microscopy using an Olympus BX43 microscope and photographed using a digital camera (Olympus XC30).

The area of newly formed bone was measured as a proportion of the total defect area and calculated as a percentage.

### Immunofluorescence

The paraffin embedded calvarial sections were deparaffinized and rehydrated in alcohol gradient (100%, 96% and 70% volume). Sections were washed and antigen unmasking was performed with sodium citrate buffer (pH 6.0). Slides were blocked with 1% bovine serum albumin (BSA) and 5% normal goat serum in phosphate buffered saline (PBS) solution and incubated for 1 h at room temperature, followed by three washes with PBS. Primary antibodies BMP, Runx-2, OPN and OCN were added in 1% BSA solution and incubated overnight at 4 °C at 1:100 dilution. The slides were washed and then incubated with the corresponding secondary antibodies diluted 1:500 (Alexa Fluor dye conjugated) in the appropriate blocking solution for 1 h at room temperature in dark. Counterstaining of nuclei was preformed with DAPI. Stained slides were mounted in fluorescence mounting medium (Sigma Chemical) and analyzed under a Leica TCS SP8 confocal microscope.

### Sample preparation for scanning electron microscopy (SEM)

The samples were mounted on conductive aluminum pin stub specimen using adhesive carbon discs on both sides. The samples were metallized with gold using a sputter coater Agar with a layer of 3 nm thickness/deposition for 3 times^50^. SEM analysis parameters were HV mode, 5–20 kV, ETD, 2 magnification order 100–300x for a general overview image and higher for surface and fracture morphology side of bones. Examination and image analysis were conducted on a FEI Quanta 250 microscope.

### Assessment of *in vivo* osteogenic markers gene expression

Bone fragments of ~0.25 cm^2^ from mouse calvaria including bone defect and substitution with CHT/GO biomaterials were collected after 72 h, 7 days, 4, 8 and 18 weeks of experiment from at least 3 individuals. Samples were collected and stored in RNA later and frozen at −80 °C until processing.

RNA was isolated from these samples using a combination of Trizol and column-based methods, due to the very low amount of material available. Briefly, bone fragments were frozen in liquid nitrogen and crushed in mortar and pestle, in 1 ml of Trizol (Life technologies, Thermo Fisher, Foster City, CA). The lysate was collected on a column available in the RNA isolation kit (InnuPrep RNA Mini Kit, AnalytikJena, Berlin, Germany). Once the RNA was purified, it was tested for integrity (BioAnalyzer 2100, Agilent Technologies, Waldbronn, Germany) and then reverse transcribed to cDNA using iScript cDNA Synthesis kit (BioRad, Hercules, CA, USA). qPCR was employed to assess the expression of specific osteogenic markers- Runx-2, OPN, OCN (LightCycler Fast Start SYBR Green, Roche, Mannheim, Germany). The osteogenic marker expression was normalized against a set of two reference genes (TATAA Binding Protein (TBP) and Glyceraldehyde phosphate dehydrogenase (GAPDH)) and qPCR data were analyzed using ∆∆Ct method, in accordance with MIQE guidelines.

### Statistical Analysis

Data was statistically processed using GraphPad Prism 3.03 software (GraphPad Software, Inc., La Jolla, CA, USA), and one-way analysis of variance, followed by a Bonferroni test. P < 0.05 was considered to indicate a statistically significant difference.

## Electronic supplementary material

Supplementary information

## References

[1] Wu S, Liu X, Yeung K, Liu C, Yang X (2014). Biomimetic porous scaffolds for bone tissue engineering. Materials Science and Engineering R.

[2] Asti A, Gioglio L (2014). Natural and synthetic biodegradable polymers: different scaffolds for cell expansion and tissue formation. Int J Artif Organs.

[3] Tadokoro M (2012). Bone morphogenetic protein-2 in biodegradable gelatin and β-tricalcium phosphate sponges enhances the *in vivo* bone-forming capability of bone marrow mesenchymal stem cells. J Tissue Eng Regen Med.

[4] Haugen HJ (2013). Porous ceramic titanium dioxide scaffolds promote bone formation in rabbit peri-implant cortical defect model. Acta Biomaterialia.

[5] Whited BM, Whitney JR, Hofmann MC, Xu Y, Rylander MN (2011). Pre osteoblast infiltration and differentiation in highly porous apatite-coated PLLA electrospun scaffolds. Biomaterials.

[6] Ben-David D (2013). Low dose BMP-2 treatment for bone repair using a PEGylated fibrinogen hydrogel matrix. Biomaterials.

[7] Vaquette C, Ivanovski S, Hamlet SM, Hutmacher DW (2013). Effect of culture conditions and calcium phosphate coating on ectopic bone formation. Biomaterials.

[8] Liu H (2013). The promotion of bone regeneration by nanofibrous hydroxyapatite/chitosan scaffolds by effects on integrin-BMP/Smad signaling pathway in BMSCs. Biomaterials.

[9] Lee JS (2014). *In vivo* study of chitosan-natural nano hydroxyapatite scaffolds for bone tissue regeneration. International Journal of Biological Macromolecules.

[10] Gao C (2014). Current Progress in Bioactive Ceramic Scaffolds for Bone Repair and Regeneration. Int. J. Mol. Sci..

[11] Cheung HY, Lau KT, Lu TP, Hui D (2007). A critical review on polymer-based bio-engineered materials for scaffold development. Composites: Part B.

[12] Peled E, Boss J, Bejar J, Zinman C, Seliktar D (2007). A novel poly(ethylene glycol)-fibrinogen hydrogel for tibial segmental defect repair in a rat model. J Biomed Mater Res A.

[13] LanLevengooda SK, Zhang M (2014). Chitosan-based scaffolds for bone tissue engineering. J. Mater. Chem. B.

[14] Almeida C.R CR (2014). Impact of 3-D printed PLA- and chitosan-based scaffolds on human monocyte/macrophage responses: Unraveling the effect of 3-D structures on inflammation. Acta Biomaterialia.

[15] Hermenean, A., Dinescu, S., Ionita, M. &. Costache, C. The Impact of Graphene Oxides on Bone Regeneration Therapies, in *Advanced Techniques in* Bone *Regeneration* (eds Zorzi, A. R. & de Miranda, J. B.) 151–167 (InTech, 2016).

[16] La W.G WG (2013). Delivery of a therapeutic protein for bone regeneration from a substrate coated with graphene oxide. Small.

[17] Nayak TR (2011). Graphene for controlled and accelerated osteogenic differentiation of human mesenchymal stem cells. ACS Nano.

[18] Li D, Kaner RB (2008). Materials science. Graphene-based materials. Science.

[19] Hong J (2012). Graphene multilayers as gates for multi-week sequential release of proteins from surfaces. ACS Nano.

[20] Wu C (2015). Graphene oxide modified beta-tricalcium phosphate bioceramics stimulate *in vitro* and *in vivo* osteogenesis. Carbon.

[21] Pandele AM (2013). Preparation and *in vitro*, bulk, and surface investigation of chitosan/graphene oxide composite films. Polymer Composites.

[22] Pandele AM (2014). Synthesis, characterization, and *in vitro* studies of graphene oxide/chitosan-polyvinyl alcohol films. Carbohydrate Polymers.

[23] Ionita M (2015). Synthesis, characterization and *in vitro* studies of polysulfone/graphene oxide composite membranes. Composites: Part B.

[24] Dinescu S (2014). *In vitro* cytocompatibility evaluation of chitosan/graphene oxide 3D scaffold composites designed for bone tissue engineering. Biomed.Mater.Eng..

[25] Ionita M (2016). Gelatin–poly(vinyl alcohol) porous biocomposites reinforced with graphene oxide as biomaterials. J.Mater.Chem. B..

[26] Ye JH (2011). Critical-size calvarial bone defects healing in a mouse model with silk scaffolds and SATB2-modified iPSCs. Biomaterials.

[27] Qureshi AT (2015). Photoactivated miR-148b–nanoparticle conjugates improve closure of critical size mouse calvarial defects. Acta Biomaterialia.

[28] Deng Y (2013). The role of miR-31-modified adipose tissue-derived stem cells in repairing rat critical-sized calvarial defects. Biomaterials.

[29] Liu X, Rahaman MN, Fu Q (2013). Bone regeneration in strong porous bioactive glass (13-93) scaffolds with an oriented microstructure implanted in rat calvarial defects. Acta Biomaterialia.

[30] Wang H (2015). Three-dimensional zinc incorporated borosilicate bioactive glass scaffolds for rodent critical-sized calvarial defects repair and regeneration. Colloids and Surfaces B: Biointerfaces.

[31] Dong (2015). A Dual Role of Graphene Oxide Sheet Deposition on Titanate Nanowire Scaffolds for Osteo-implantation: Mechanical Hardener and Surface Activity Regulator. Scientific reports.

[32] Pandele MA (2017). Porous Chitosan/Graphene Oxide Biocomposites for Tissue Engineering. Polymer Composites.

[33] Venkatesan J, Vinodhini PA, Sudha PN, Kim SK (2014). Chitin and chitosan composites for bone tissue regeneration. Adv. Food Nutr. Res.

[34] Lee JH (2015). Enhanced Osteogenesis by Reduced Graphene Oxide/Hydroxyapatite Nanocomposites. Scientific Reports.

[35] La WG (2014). Delivery of bone morphogenetic protein-2 and substance p using graphene oxide for bone regeneration. Int. J.Nanomedicine.

[36] Shahriarpanah S, Nourmohammadi J, Amoabediny G (2016). Fabrication and characterization of carboxylated starch-chitosan bioactive scaffold for bone regeneration. International Journal of Biological Macromolecules.

[37] Di Martino A, Sittinger M, Risbud MV (2005). Chitosan: a versatile biopolymer for orthopaedictissue-engineering. Biomaterials.

[38] Rodríguez-Vázquez, M., Vega-Ruiz, B., Ramos-Zúñiga, R., Saldaña-Koppel, D. A. & Quiñones-Olvera, L. F. Chitosan and Its Potential Use as a Scaffold for Tissue Engineering inRegenerative Medicine. *BioMed Research Internationa*l 15 pages, http://dx.doi.org/10.1155/2015/821279 (2015). 10.1155/2015/821279 PMC4609393 26504833

[39] Jones AC (2009). The correlation of pore morphology, interconnectivity and physical properties of 3D ceramic scaffolds with bone ingrowth. Biomaterials.

[40] Balta C (2016). Homeostasis of blood parameters and inflammatory markers analysis during bone defect healing after scaffolds implantation in mice calvaria defects. Romanian Biotechnological Letters.

[41] Hartman EHM, Vehof JWM, De Ruijter JE, Spauwen PHM, Jansen JA (2004). Ectopic bone formation in rats: the importance of vascularity of the acceptor site. Biomaterials.

[42] Osta B, Lavocat F, ELjaafari A, Miossec P (2014). Effects of interleukin-17A on osteogenic differentiation of isolated human mesenchymal stem cells. Frontiers in Immunology.

[43] Rosen V (2009). BMP2 signaling in bone development and repair. Cytokine & Growth Factor Reviews.

[44] Komori T (2011). Signaling Networks in RUNX2-Dependent Bone Development. Journal of Cellular Biochemistry.

[45] Wu M, Chen G, Li YP (2016). TGF-β and BMP signaling in osteoblast, skeletal development, and bone formation, homeostasis and disease. Bone Research.

[46] Ma X, Li Y, Wang W, Ji Q, Xia Y (2013). Temperature-sensitive poly (Nisopropylacrylamide)/graphene oxide nanocomposite hydrogels by *in situ* polymerization with improved swelling capability and mechanical behavior. Eur. Polym. J..

[47] Kandiah K, Muthusamy P, Mohan S, Venkatachalam R (2014). TiO2–graphene nanocomposites for enhanced osteocalcin induction. Materials Science and Engineering C.

[48] Herberg S (2014). Cray, Inkjet-based biopatterning of SDF-1β augments BMP-2-induced repair of critical size calvarial bone defects in mice. Bone.

[49] Pigossi SC (2015). Bacterial cellulosehydroxyapatite composites with osteogenic growth peptide (OGP) or pentapeptide OGP on bone regeneration in critical-size calvarial defect model. J. Biomed. Mater. Res. Part A.

[50] Ludwig, R. *Scanning Electron Microscopy: Physics fo Image Formation and Microanalysis* (Springer, 1998).

