# Supplementary material for: Chitosan-Graphene Oxide 3D scaffolds as Promising Tools for Bone Regeneration in Critical-Size Mouse Calvarial Defects
Source: Sci Rep. 2017 Nov 30;7:16641. doi: 10.1038/s41598-017-16599-5 (PMC5709492; doi:10.1038/s41598-017-16599-5)
Supplement: Supplementary file 1 — Supplementary information [file 41598_2017_16599_MOESM1_ESM.pdf]

SUPPLEMENTARY MATERIAL

Chitosan-Graphene Oxide 3D scaffolds as Promising Tools for Bone Regeneration in Critical-Size Mouse Calvarial Defects

Anca Hermenean[1,2*],Ada Codreanu[1], Hildegard Herman[2], Cornel Balta[2], Marcel Rosu[2], Ciprian Mihali[2], Alexandra Ivan[3], Sorina Dinescu[4], Mariana Ionita[5], Marieta Costache[4]

[1]Department of Histology, Faculty of Medicine, Vasile Goldis Western University of Arad, 86 Rebreanu, 310414 Arad, Romania
[2]Department of Experimental and Applied Biology, Institute of Life Sciences, Vasile Goldis Western University of Arad, 86 Rebreanu, 310414 Arad, Romania
[3]Department of Functional Sciences, Victor Babes University of Medicine and Pharmacy, 300041 Timisoara
[4]Department of Biochemistry and Molecular Biology, University of Bucharest, 91-95 Splaiul Independentei, 050095 Bucharest, Romania
[5]Advanced Polymer Materials Group, University Politehnica of Bucharest, CaleaVictorie 147, Bucharest 010737, Romania

* Coresponding author: Department of Histology, Faculty of Medicine, Vasile Goldis Western University of Arad, 86 Rebreanu, 310414 Arad, Romania,
e-mail address: anca.hermenean@gmail.com

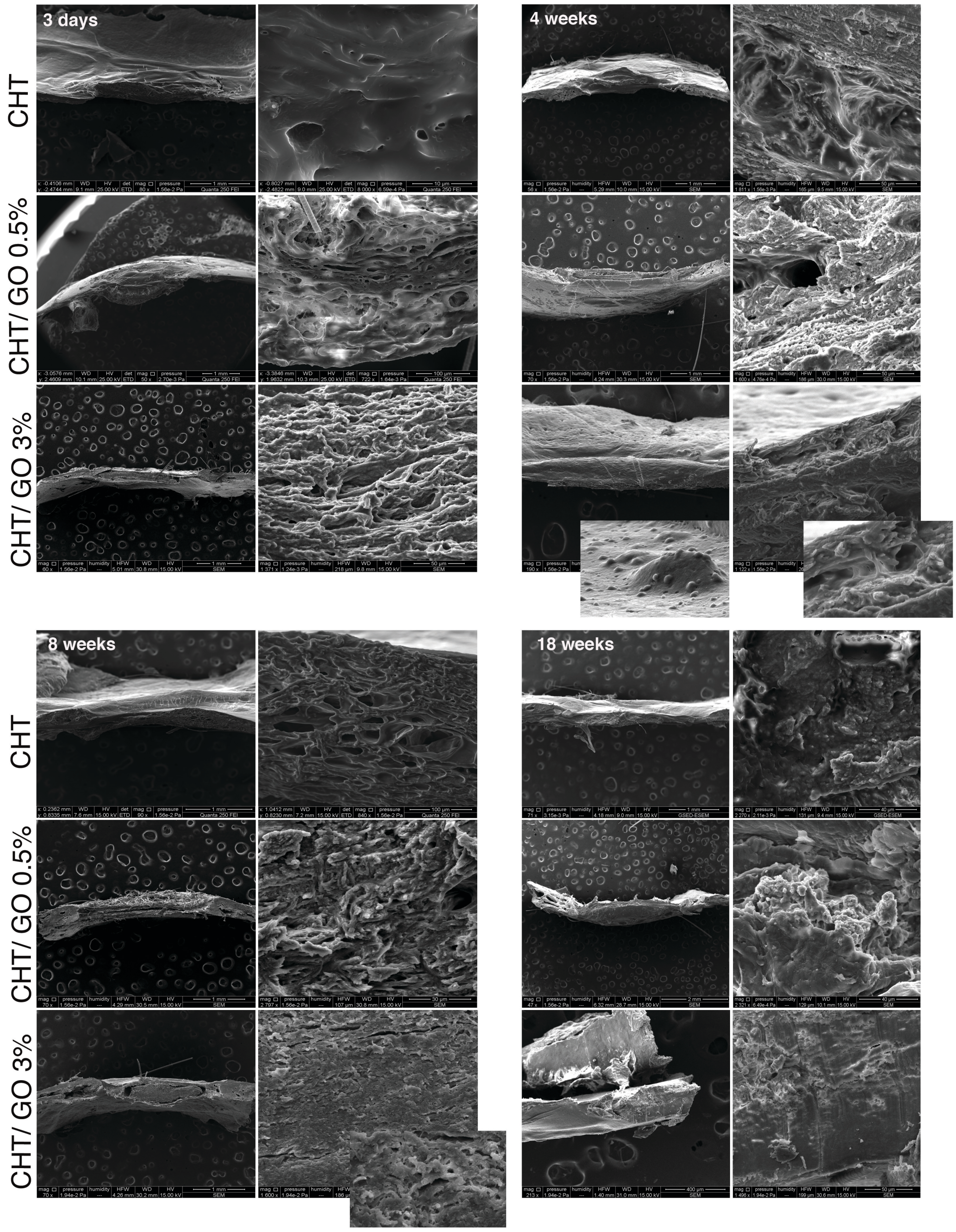

Figure 1: SEM micrographs of the *in vivo* bone samples taken after 72 hours, 4, 8 and 18 weeks after CHT, CHT/GO 0.5 wt% and CHT/GO 3.0 wt.% scaffold implantation (*all time-intervals*)

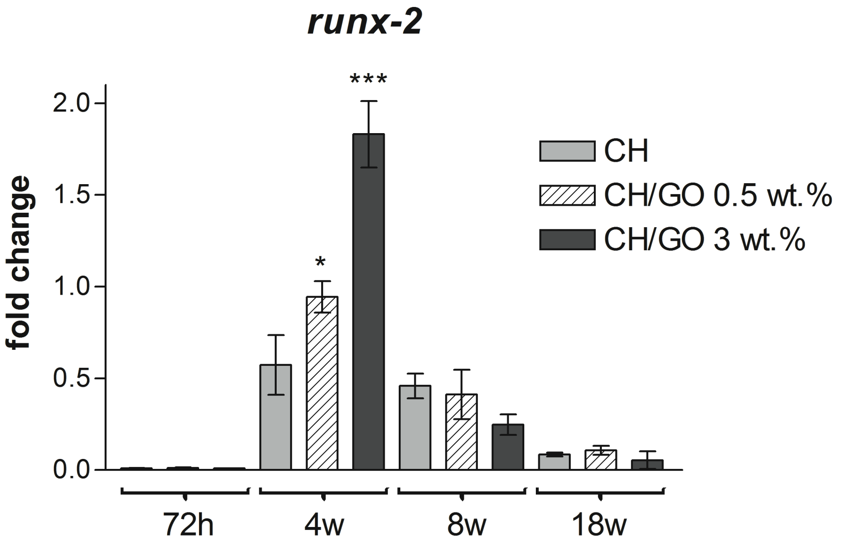

Figure 2: mRNA expression of Runx-2 at 72h, 4 weeks, 8 weeks and 18 weeks after CHT, CHT/GO 0.5 wt% and CHT/GO 3.0 wt.% scaffold implantation

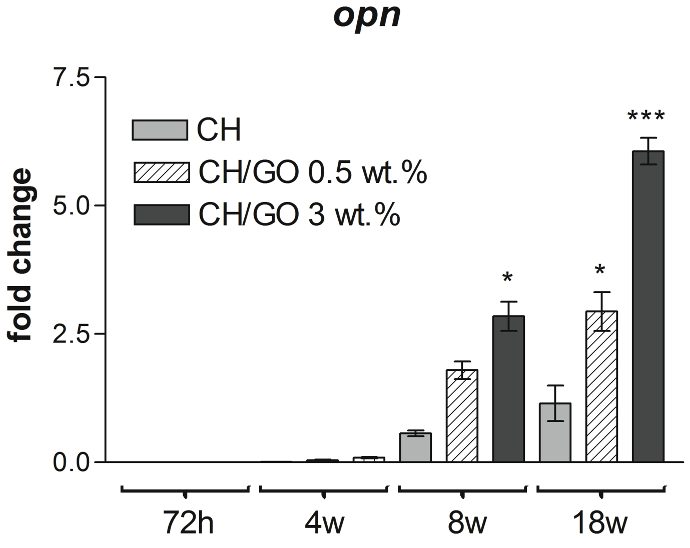

Figure 3: mRNA expression of OPN at 72h, 4 weeks, 8 weeks and 18 weeks after CHT, CHT/GO 0.5 wt% and CHT/GO 3.0 wt.% scaffold implantation

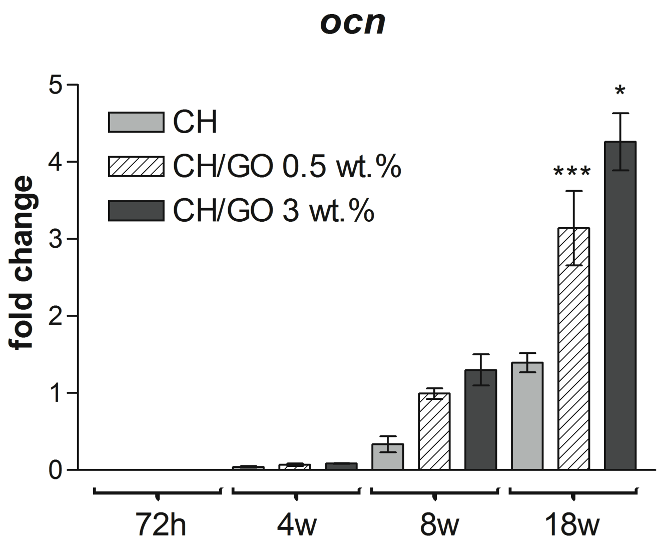

Figure 4: mRNA expression of OCN at 72h, 4 weeks, 8 weeks and 18 weeks after CHT, CHT/GO 0.5 wt% and CHT/GO 3.0 wt.% scaffold implantation

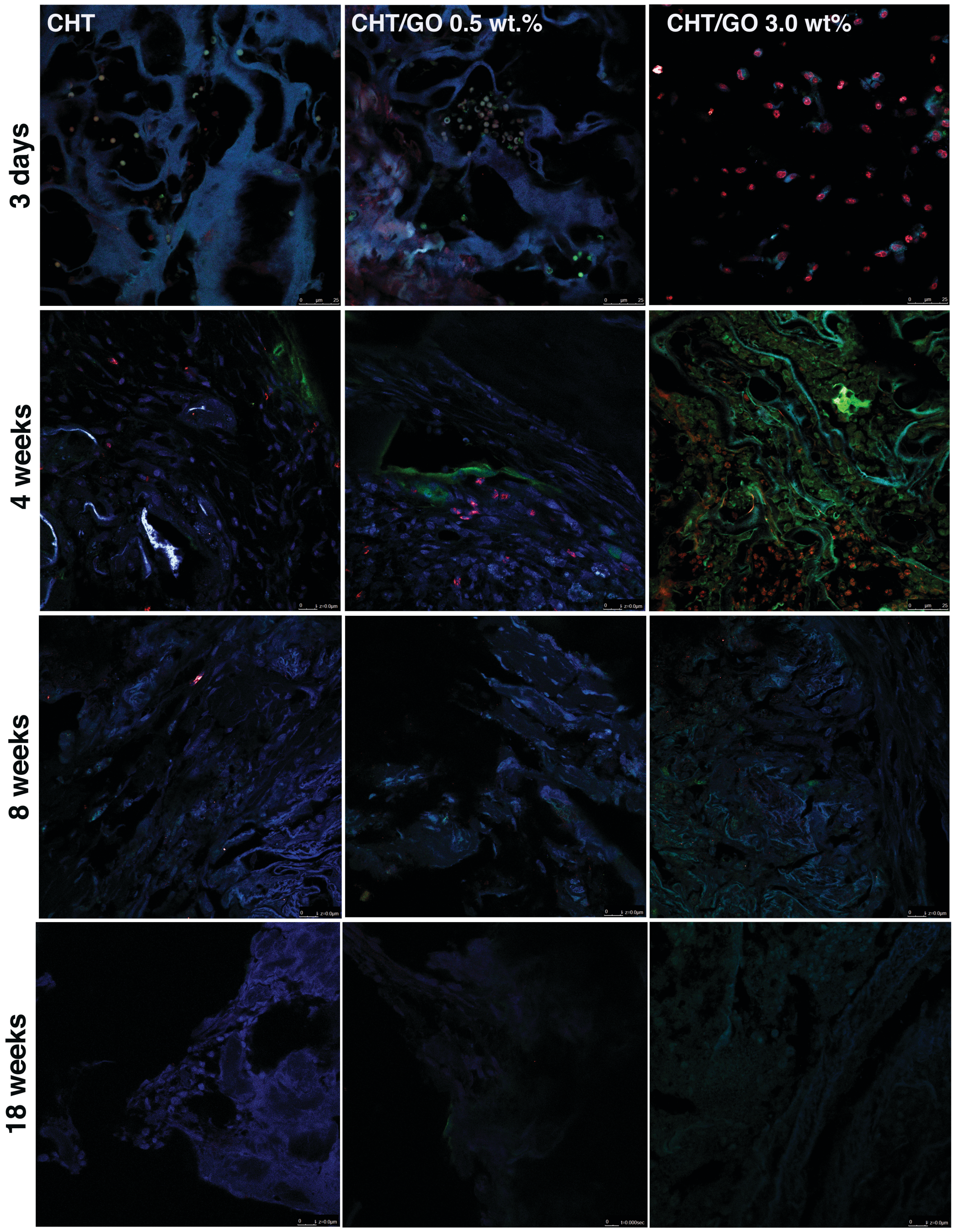

Figure 5: Immunohistochemical expression of BMP-2 and Runx-2 at 72h, 4 weeks, 8 weeks and 18 weeks after CHT, CHT/GO 0.5 wt% and CHT/GO 3.0 wt.% scaffold implantation

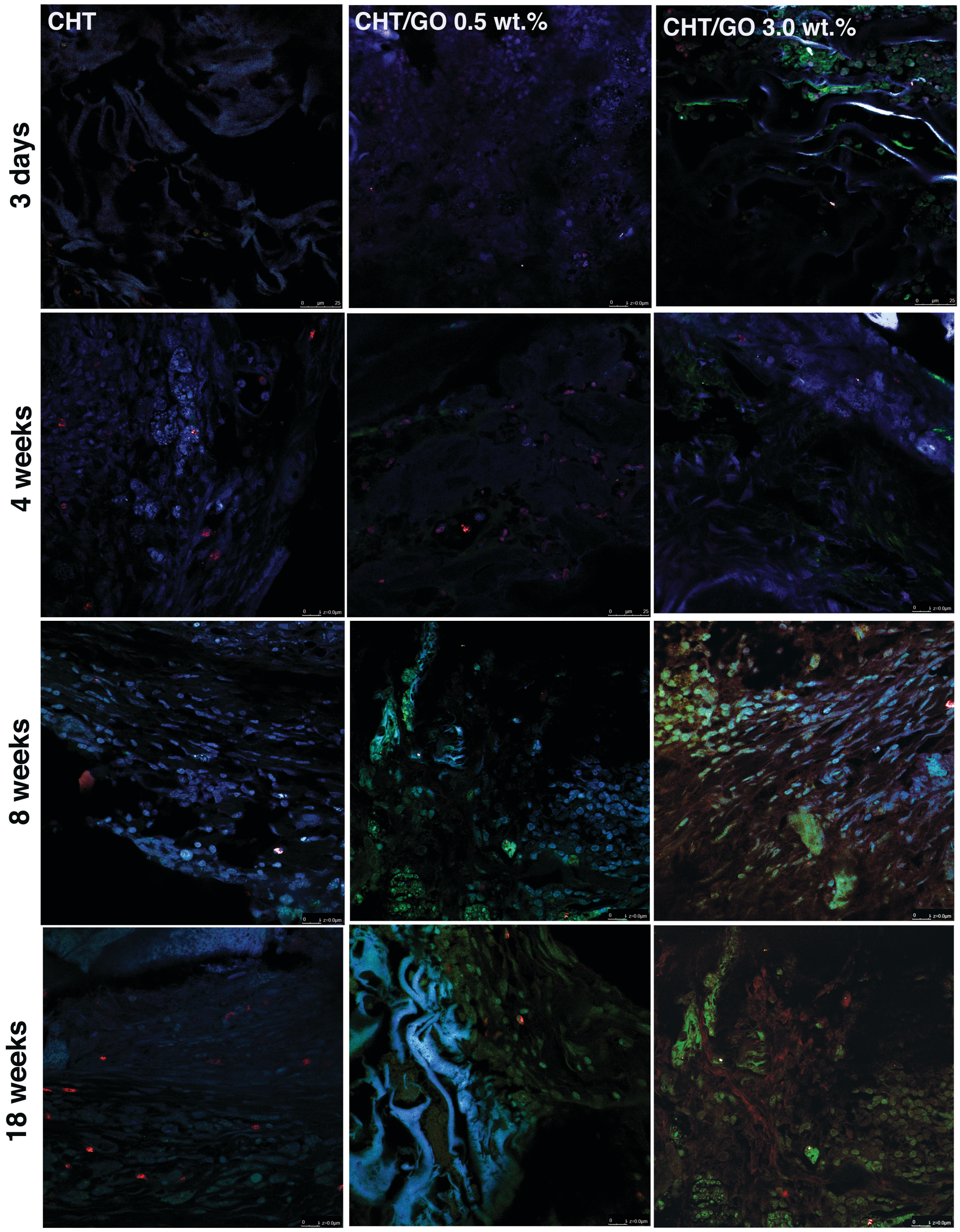

Figure 6: Immunohistochemical expression of OPN and OCN at 72h, 4 weeks, 8 weeks and 18 weeks after CHT, CHT/GO 0.5 wt% and CHT/GO 3.0 wt.% scaffold implantation
